# A National Census of Birth Weight in Purebred Dogs in Italy

**DOI:** 10.3390/ani7060043

**Published:** 2017-05-30

**Authors:** Debora Groppetti, Alessandro Pecile, Clara Palestrini, Stefano P. Marelli, Patrizia Boracchi

**Affiliations:** 1 Department of Veterinary Medicine, Faculty of Veterinary Medicine, Università degli Studi di Milano, via Celoria 10, 20133 Milan, Italy; alessandro.pecile@unimi.it (A.P.); clara.palestrini@unimi.it (C.P.); stefano.marelli@unimi.it (S.P.M.); 2 Department of Clinical Sciences and Community Health, Università degli Studi di Milano, via Vanzetti 5, 20133 Milan, Italy; patrizia.boracchi@unimi.it

**Keywords:** birth weight, dog, morphometry

## Abstract

**Simple Summary:**

Birth weight is a key factor for neonatal mortality and morbidity in most mammalian species. The great morphological variability in size, body weight and breed, as well as in skeletal and cranial conformation makes it challenging to define birth weight standards in dogs. A total of 3293 purebred pups were surveyed to study which maternal aspects can determine birth weight considering head and body shape, size, body weight and breed in bitches, as well as litter size and sex in pups. In our sample, multivariate analysis outcomes suggested that birth weight and litter size were directly proportional to maternal size. The maternal body shape influenced both birth weight and litter size, whereas the maternal head shape had impact only on birth weight. Sex differences in birth weight were found. Birth weight and litter size also varied among breeds. The results of the present study could have practical implications allowing one to identify pups in need of admission to intensive nursing care, as occurs in humans. A deeper knowledge of the factors that significantly influence birth weight could positively affect the canine breeding management helping to prevent and reduce neonatal mortality.

**Abstract:**

Despite increasing professionalism in dog breeding, the physiological range of birth weight in this species remains unclear. Low birth weight can predispose to neonatal mortality and growth deficiencies in humans. To date, the influence of the morphotype on birth weight has never been studied in dogs. For this purpose, an Italian census of birth weight was collected from 3293 purebred pups based on maternal morphotype, size, body weight and breed, as well as on litter size and sex of pups. Multivariate analysis outcomes showed that birth weight (*p* < 0.001) and litter size (*p* < 0.05) increased with maternal size and body weight. Birth weight was also influenced by the maternal head and body shape, with brachycephalic and brachymorph dogs showing the heaviest and the lightest pups, respectively (*p* < 0.001). Birth weight decreased with litter size (*p* < 0.001), and male pups were heavier than females (*p* < 0.001). These results suggest that canine morphotype, not only maternal size and body weight, can affect birth weight and litter size with possible practical implications in neonatal assistance.

## 1. Introduction

Birth weight has an important effect on fetal and neonatal health in humans. Due to their immature development and adaptive postnatal failure, underweight babies are prone to potential complications, especially impaired thermoregulation and hypoglycemia [1]. Therefore, they are susceptible to mortality and morbidity, developing cerebral palsy, hyaline membrane disease, apnea, intracranial hemorrhage, sepsis, retrolental fibroplasia, growth and neurocognitive deficiencies [2]. Low birth weight can result from either a short gestation period or retarded intrauterine growth (or a combination of both) [2] as reported for humans and animals of many polytocous species, including dogs [3]. Based on evidence of embryo transfer studies in the human, horse and sheep, the intrauterine environment in which the fetus develops seems to exert a profound effect on birth weight, suggesting a central maternal role in determining the birth weight [4]. Anthropometric parameters, mainly head circumference, provide an indirect measure of low birth weight in babies and may thus be of prognostic significance [5,6]. Moreover, maternal factors such as height and weight of the woman are positively related with term fetal weight [2,7,8]. The same implication could be assumed in the canine species.

Due to the wide phenotypic variability among breeds, dogs offer a unique opportunity to study correlations between morphology and birth weight. In fact, there are 337 breeds of domestic dogs (*Canis familiaris*) recognized by the Fédération Cynologique Internationale (FCI). Bench standard defines the ideal characteristics for each breed including size (height at withers), body weight and morphometry of the adult dogs, while no specific information on the birth weight is provided. To date, despite a large number of studies on the puppy growth chart [9], the influence of the morphotype on birth weight has never been studied in dogs. The cephalic index or cranial index is the ratio between maximum width and length of the skull of an organism (human or animal). This index is used to classify animals into three groups: brachycephalic, mesocephalic and dolichocephalic [10]. Similarly, the relationship between height at withers and thoracic conformation determines the division of dog breeds into brachymorph, mesomorph, dolichomorph and anacholicomorph type [11,12].

The pursuit of an optimal model to classify the purebred dog still represents an important goal for scientific purposes. This study for the first time correlates birth weight with phenotypic aspects in purebred dogs, namely considering the impact of different morphometric characteristics. The objective of the study was to detect which parameters can influence the birth weight of pups and the litter size among maternal morphotype (head and body shape), size (height at withers) and body weight (BW). An exploratory investigation of the influence of breed on birth weight was conducted in selected groups of dogs with the same morphotype.

## 2. Materials and Methods 

This study is based on data collected through an on-line questionnaire administered to Italian dog breeders from February 2014–September 2015 in the context of a national census promoted by the Università degli Studi di Milano in collaboration with the Ente Nazionale della Cinofilia Italiana (ENCI) to register the birth weight in the Italian purebred dog population. Participation in the questionnaire was freely decided by the breeders. In this case, the approval of the Ethics Committee does not apply.

A large-scale prospective study to survey the birth weight (body weight of pups at birth (bBW)) of 3293 pups from a sample population of 588 purebred bitches of 99 breeds from a population of 154,195 dogs in Italy (see Appendix A) was performed (Table 1). Litter size, breed, as well as birth weight and sex of pups were recorded by the census. Maternal data, such as head shape (cranial index), body shape, size (height at withers) and body weight, were taken from FCI, ENCI and kennel clubs.

### 2.1. Definitions

The Total Cephalic Index (TCI) is the ratio between the cranium width and the head length (tip of the nose-tip of the occiput). Based on their head shape, dogs were classified as brachycephalic (TCI > 50), mesocephalic (TCI = 50) and dolichocephalic (TCI < 50) [11]. The Corporal Index (CI) is the ratio between the length of the body (point of shoulder-ischiatic tuberosity) and the thoracic girth. According to their body shape, dogs were divided into: brachymorph (CI = 60–70), mesomorph (CI = 71–84), dolichomorph (CI = 85–100) and anacholicomorph [11,12]. Anacholicomorph, a term derived from Greek, means short legged: basset-like proportion [11]. Dogs were also categorized into groups according to maternal size, i.e., height at withers (<20 cm: toy; 20 cm ≤ small ≤ 40 cm; 40 cm < medium ≤ 65 cm; >65 cm: large) [13] and maternal body weight (<5 kg; 5 kg ≤ BW ≤ 10 kg; 10 kg < BW ≤ 25 kg; 25 kg < BW ≤ 45 kg; >45 kg) [14]. The sex of pups was recorded at birth and stated as undefined when it was ambiguous or pups were malformed. We included both live and stillborn pups in the database.

### 2.2. Statistical Analysis

The distribution of birth weight and number of pups according to the maternal characteristics mentioned above was synthesized by the following indices: minimum, first quartile (1st Q), median, mean, third quartile (3rd Q) and maximum.

The relationship between birth weight of the pups (response variable) and litter size, maternal characteristics and sex of pups (explicative variables) was evaluated by linear mixed regression model. Litter size, maternal characteristics and sex of pups were considered as fixed effects. The correlation among pups from the same litter was accounted for including in the model the mother’s identification code as a random effect. The categorical maternal characteristics and sex of pups were included in the regression model as dummy variables. For a categorical variable with *k* categories, one of the categories is considered as the “reference”, and *k-1* dummy variables are generated to compare the mean of the response variable in each category with the mean of the response variable in the reference category. The number of pups per litter was included in the regression model in its original measurement scale. Residual analysis suggested the use of the logarithmic transformation of the birth weight. After logarithmic transformation, the regression coefficients can be related to the geometric mean of the response variable (i.e., the mean of the logarithm of birth weight is the geometric mean of birth weight rather than the usual arithmetic mean of the birth weight). For categorical variables, the exponent of the regression coefficient of each dummy variable was the estimate of the ratio between the geometric mean of birth weight of the category represented by the dummy variable and the geometric mean of the birth weight of the reference category. For litter size, the exponent of the regression coefficient was the estimate of the ratio between the geometric mean of the birth weight for each of two consecutive litter size values.

The null hypothesis of the regression coefficient equal to 0 for fixed effects was tested by the *t* statistic. To perform adequate inference procedures, Satterthwaite’s approximation of the degree of freedom of the *t* statistic was applied. The relationship between the litter size (response variable) and the maternal characteristics (explicative variables) was evaluated by a generalized linear model with Poisson error. As the considered maternal characteristics are categorical, each characteristic dummy variable was generated as previously described. In this generalized linear model, the exponent of the regression coefficient of each dummy variable was the estimate of the ratio between the mean litter size of the category represented by the dummy variable and the mean litter size of the reference category. The null hypothesis of each regression coefficient equal to 0 was tested by the Wald statistic. For both Poisson and linear mixed regression models, the following results related to the explicative categorical variables are reported: model estimated mean of the response variable for each category; ratio between the estimated mean of the response variable in each category and the estimated mean of the response variable in the reference category; and the 95% confidence interval of the ratio. For the numerical explicative variable, the following results are reported: model estimated mean of the response variable for the lowest value of the explicative variable and the increase of the mean of the response variable for a one-unit increase of the explicative variable. For both the Poisson and linear mixed model, the effect of each explicative variable was evaluated firstly by univariate analysis, then a multivariable regression model was used to evaluate the joint role of all of the explicative variables. The authors consider the maternal body weight and size as correlated, so two alternative multivariable regression models were performed including weight and size, respectively. A parsimonious final model was obtained by the stepwise selection procedure.

An exploratory analysis was performed to evaluate the association between breeds and birth weight and between breeds and litter size in dogs sharing the same morphotype. To obtain reliable results, only breeds represented by at least 15 litters were considered.

Statistical significance was accepted at *p* < 0.05.

The analysis was performed by the R Core Team (2016) software; R: A language and environment for statistical computing; R: Foundation for Statistical Computing, Vienna, Austria. ISBN 3-900051-07-0 [15]; package lme4 for bBW [16]; and the glm function for the number of pups.

## 3. Results

The distribution of pup number and birth weight according to combinations of maternal morphotype, size and body weight of dogs in the study is summarized in Table 2.

### 3.1. Birth Weight

The birth weight ranged from 40–1250 g. The lightest pup was a German Spitz (Pomeranian-Zwergspitz) that died within 24 h after birth; its surviving littermate weighed 124 g. The heaviest pup was from a Mastino Napoletano dog that delivered eight healthy pups. 

For statistical purposes, 45 pups whose data were incomplete were excluded from our investigation, thus including a total of 3248 pups.

In univariate analysis, maternal head shape significantly influenced the mean birth weight of brachycephalic dogs when compared to mesocephalic dogs (Table 3). Similarly, in brachymorph dogs, birth weight was related to maternal body shape when compared to mesomorph dogs. In the remaining morphological categories, no statistical differences in bBW were observed with respect to brachymorph.

The mother’s size was directly related to the bBW. Similar results were obtained considering the mother’s body weight. Concerning the relationship between the logarithm of the bBW and the litter size, no evidence for a non-linear effect was found. The estimated bBW increased with the increase of the number of pups per litter. To clarify this result, the effect of litter size on birth weight was adjusted for mother’s size. In this case, the impact of litter size was not statistically significant and inversely proportional to birth weight (mean ratio 0.99, *p* > 0.3).

When maternal head shape, body shape, body weight, number of pups per litter and the sex of pups were jointly considered, the mother’s head shape did not contribute significantly to the bBW (*p* = 0.0558) and was excluded from the final regression model by the stepwise procedure. Concerning the body shape, the mean bBW of brachymorph dogs was significantly lower than that of all other categories. As already mentioned, the average bBW increased with the decreasing of the litter size. Results of the final regression model are reported in Table 4.

When the mother’s size was considered instead of the mother’s weight, all variables showed a significant contribution (Table 5). The mean bBW of brachycephalic dogs was significantly greater than that of mesocephalic and dolichocephalic dogs. Concerning maternal body shape, the mean bBW of anacholicomorph dogs was significantly greater than that of brachymorph dogs, and no significant differences were found among the other maternal body shape categories. The results for litter size were similar to those reported above.

The contribution of maternal body weight and size to the model was F = 341.32, *p* < 0.0001 and F = 273.47, *p* < 0.0001, respectively.

### 3.2. Litter Size

Litter size ranged from 1–14 pups with the largest litter delivered by a Rhodesian ridgeback dog. In univariate analysis, the mother’s head shape was related to litter size with mesocephalic dogs delivering litters at a mean 1.2-times more numerous than brachycephalic ones (Table 6). Similarly, mesomorph dogs had litters at a mean more numerous than brachymorph dogs. Litter size was directly proportional to maternal size. Likewise, the litter size increased proportionally to the maternal body weight.

When maternal head shape, body shape and body weight were jointly considered, the contribution of head shape was not statistically significant, and the final model excluded this variable (Table 7). The maternal body shape influenced litter size with brachymorph dogs delivering the lowest number of pups. The mean number of pups increased with the increase of the maternal BW. Similarly, when maternal size was considered in the model instead of BW, the head shape did not contribute significantly to litter size and was not included in the final model (Table 8). However, the impact of maternal body shape on the number of pups showed a minor contribution with a difference in litter size only emerging between brachymorph and anacholicomorph dogs. The mean number of pups increased with the increasing of the maternal size.

### 3.3. Breed

According to our inclusion criteria, five breeds were considered for exploratory analysis: German shepherd, golden retriever, Jack Russel terrier, Labrador retriever and West Highland white terrier (WHWT) (Table 9). German shepherd, golden retriever and Labrador retriever belong to mesocephalic, mesomorph, medium sized, 25–45-kg weighing dogs. Jack Russel terrier and WHWT belong to mesocephalic, mesomorph, small-sized, 5–10-kg weighing dogs.

The bBW was lower in WHWT than in Jack Russel terrier (*p* < 0.05), while no significant differences were recorded among the other breeds. Few differences were found between mean bBW of German shepherd, golden retriever and Labrador retriever (reference): the estimated mean ratio was 0.9 and 1.03, respectively.

The ratio between mean litter size of Labrador retriever and golden retriever was 0.77 (*p* = 0.01). The ratio between mean litter size of German shepherd and golden retriever was 0.85 (*p* = 0.12). Only the comparison between Labrador and golden retriever was statistically significant. No significant differences in mean litter size between Jack Russel terrier and WHWT were recorded (mean ratio = 0.951).

### 3.4. Sex of Pups

In our samples, 1559 pups were females and 1665 males. In the remaining 24 pups, the sex was not defined, as it was ambiguous or they were malformed. In univariate analysis, mean bBW of males was greater than mean bBW of females (mean ratio = 1.04, *p* < 0.001; Table 3). The mean bBW of pups with undefined sex was lower than mean bBW of females (mean ratio = 0.86, *p* < 0.001). These results were confirmed by multivariate analysis (*p* < 0.001; Table 4 and Table 5).

## 4. Discussion

Despite its relevant impact on neonatal and adult health, deep knowledge of factors affecting birth weight in dogs is still lacking. As already noted, low birth weight in pups, as well as in babies, kittens and piglets, leads to higher risk of neonatal morbidity and mortality compared with normal weight littermates [17,18,19]. Mortality of pups attributed to low birth weight is reported from 1.4% [20] to 2.1% [21]. In large-sized breeds, birth weight in dogs dying during the first week after birth was 100 g lower than in surviving pups [22,23]. Moreover, pup weight at birth has a significant influence on the outcome of parturition [24], and being oversized in pups may be responsible for uterine inertia and consequent fetal distress [25,26]. Pups being oversized in the case of singleton pregnancy, as well as disproportion between maternal pelvic and pup head dimensions are known to be predisposing factors to dystocia since more uterine force is needed to expel these pups [25]. Namely, dystocia is reported to occur more likely in some canine breeds and morphotypes, with increasing cranial circumference of the pups, that is in brachycephalic dogs [24,26,27,28,29,30].

Theoretically, in all mammalian species, there is an ideal range of birth weight associated with eutocic parturition and neonatal well-being [4]. To date, due to a wide morphological and morphometric variability within canine breeds, no criteria are available to recognize which range of birth weight is to be considered physiological. Even the present study has no claim to provide a birth weight cut-off for each breed, rather to investigate associations among birth weight, litter size and morphology by an original canine classification. Although the effect of maternal phenotype on birth weight was investigated by a multivariate regression model, our result cannot be used for predictive aims. Indeed, a suitable predictive model would require a very large population with independent case series for model validation. To the authors’ knowledge, this is the first study evaluating the influence of maternal morphotype, namely head and body shape and not only size and weight, on pup birth weight and number. Given that studies on the average weight of purebred pups at birth are few and based on a small scale, a thorough comparison with the available literature is not possible. However, the birth weights of the German shepherd, Labrador retriever and Rottweiler pups in our sample were similar to those previously described [31]. 

The body weight was reported to vary up to 40-times among adult dogs from different sizes and breeds, while it was only 10-times different at most among pups at birth [32]. Our results showed a greater range of birth weight than those reported by Fiszdon et al. (2009) with the thinnest pup about 31-times lighter than the heaviest one [32]. This aspect can be justified by both a different sample size of our study (*n* = 501 versus *n* = 3293 pups) and our inclusion of either live or stillborn pups. Severely underweight pups are not likely to survive. However, the relation between birth weight and neonatal mortality has not been investigated, beyond the aim of the present study. 

Data shown in this study are from a census, so they do not represent the registered database of ENCI during the same period (see Appendix A). Being that participation in the census was based on the voluntary participation of breeders, a potential bias of our sample in relation to the distribution of the whole canine Italian population is possible. Moreover, a possible ‘kennel effect’ on birth weight and litter size of dogs from the same breeder (Table 1) should not be neglected, as well as the involvement of the bloodlines.

In our canine population, the maternal head shape had a significant impact on birth weight of pups when morphotype, litter size and sex of pups were considered together with maternal size. Brachycephalic dogs had the heaviest pups. These data are consistent with studies reporting an association between low birth weight and small head circumference at birth in babies [6,33]. However, the head shape contribution was not significant when the same variables (morphotype, litter size and sex of pups) were considered together with maternal body weight. We speculate that maternal body weight may have a more powerful impact on birth weight than size. Conversely, litter size was not affected by the head shape.

The maternal body shape influenced significantly both birth weight and litter size with brachymorph dogs delivering the lightest pups and a lower number of pups than anacholicomorph ones. Studies in humans highlighted the importance of maternal phenotype influence on birth weight, indicating that weight at birth is attributable to maternal anthropometry differences and not to maternal size variability alone [34]. 

Maternal size and body weight were directly proportional to both birth weight and litter size in our sample. The same observation was reported in cats with birth weight increasing as maternal weight and height increased [35]. Similarly, observational epidemiological studies have revealed that both maternal height and weight are associated with birth weight in babies [7]. These associations have been interpreted based on a mechanistic assumption that maternal dimension sets a physical constraint on the intrauterine environment that affects fetal growth [7].

A limit of the present study is the lack of data on the real maternal body weight, body condition score, gestational weight gain and caloric intake of the dogs included in the census. Therefore, we cannot exclude that the nutritional status of bitches may also affect the birth weight of pups as described in humans [36,37]. Moreover, maternal size and weight were taken from FCI, ENCI and kennel clubs and not directly recorded by the questionnaire. Although the used classifications are reliable [13,17], a partial loss of information on the relationship between these two variables and bBW or litter size could be possible. Finally, a possible bias on birth weight recording is intrinsic in a study based on data directly collected by the owner.

As expected on the basis of the literature, litter size was inversely proportional to birth weight, with weight reduction for each additional pup per litter [26,35]. 

A significant sex difference in birth weight was recorded, with male pups being the heaviest. Data reported in literature on this topic are conflicting. Some authors have found no difference on birth weight between male and female pups [23,26]. On the contrary, other studies have shown an increased birth weight in male compared with female pups [38,39], as described in humans and sheep [2,4]. These heterogeneous results could be due to different sample sizes and different distributions of dog’s morphologic characteristics in case series. A comparison of results should be performed after taking into account litter size, maternal weight and morphotype in a multivariate analysis. 

As previously observed, ambiguous or malformed pups resulted in lighter birth weight than healthy ones [40]. In humans, congenital malformations seem to be the most important factor that determines low birth weight [41].

Heritability for body weight at birth has been demonstrated in boxers [42]. A significant breed-dependent difference in birth weight and litter size among breeds of the same size and weight was recorded in our sample, even though only five breeds have been compared. These data suggest a non-negligible role of the breed, not only size, weight and morphotype, in determining birth weight. However, the breed influence should be further investigated to be verified in very large datasets.

## 5. Conclusions

There is strong evidence that birth weight results from a complex interaction between genetic and environmental factors of parental, placental and fetal origin in humans [43]. Due to some above-mentioned limitations, besides the lack of paternal information, the outcomes of this survey should be generalized with caution, as it represents a definite sample of pedigree dog population in Italy. Studies in human reported that paternal birth weight and height are significant and independent predictors of birth weight in offspring [44,45], although maternal factors make bigger contributions to babies’ birth weight [46]. This large-scale study provides evidence that canine morphotype, not only maternal size and body weight, together with breed are involved in determining birth weight and litter size. Results of the present study have concrete implications in canine neonatal practice allowing one to deepen the knowledge of factors that significantly influence variation in birth weight and to identify pups in need of admission to intensive nursing care.

## Figures and Tables

**Table 1:** Distribution of the bitches based on their breed, morphotype, size, body weight and the corresponding number of litters and pup birth weight.

Breed	Head Shape ^a^	Body Shape ^b^	Size ^c^	BW ^d^	N_L_^e^	N_P_^f^	N_K_^g^	N_p_			bBW ^h^		
								Median	Q1 ^i^	Q3 ^l^	Median	Q1 ^i^	Q3 ^l^
Afghan Hound	D	D	4	3	2	12	2	6	5.5	6.5	500	480	562.5
Akita Inu	M	M	3	4	9	57	4	6	5	8	401	367	420
Alaskan Malamute	M	M	3	4	2	15	2	7.5	7.25	7.75	468	429	480
American Akita	M	M	4	4	2	14	2	7	7	7	576	494.5	695
American Cocker	M	M	2	3	3	17	2	6	5.5	6	200	160	220
American Staffordshire T.	M	M	3	4	1	1	1	1	1	1	500	500	500
Appenzeller Mountain dog	M	M	3	3	4	22	2	5.5	4.5	6.5	357	321.2	424.5
Australian Shepherd	M	M	3	3	7	50	6	7	6.5	8	350	320	390
Basset Hound	M	A	2	3	7	51	3	7	5.5	9	448	350	510
Beagle	M	M	2	3	4	28	4	7.5	6.5	8	303.5	278.8	352.8
Bearded Collie	M	M	3	3	1	6	1	6	6	6	395	371.8	403.2
Beauceron	D	M	4	4	1	6	1	6	6	6	535	523,8	542.5
Belgian Shepherd dog	M	M	3	4	5	31	2	7	5	8	425	387.5	460
Bernese Mountain dog	M	M	3	4	33	196	10	6	3	8	541	490	600
Bichon Havanais	M	M	2	1	8	37	3	4.5	3.75	6	195	165	215.5
Black Russian Terrier	M	M	4	5	1	8	1	8	8	8	475	457.5	491.2
Bolognese	M	M	2	1	3	10	3	3	2.5	4	137.5	130	145.5
Border Collie	M	M	3	3	13	83	10	7	5	7	350	300	378
Border Terrier	M	M	2	2	1	5	1	5	5	5	193	190	200
Borzoi	D	D	4	4	1	11	1	11	11	11	446	390.5	480.5
Boston Terrier	B	M	2	2	8	20	3	3	1.75	3	200	178	226
Bouledogue	B	B	2	3	9	46	4	5	4	6	184	150.2	235.8
Bouvier des Flandres	M	M	3	4	2	16	1	8	8	8	463.5	438.5	500
Boxer	B	M	3	4	12	82	10	8	4.75	9	449.5	400	410
Bracco Italiano	M	M	3	4	1	10	1	10	10	10	405	400	410
Brussel Griffon	B	M	2	1	2	11	2	7	7	7	120	106.5	135
Bulldog	B	B	2	3	9	32	6	3	2	4	316	280	368.8
Bullmastiff	B	M	4	5	3	22	3	7	6	8.5	597.5	579.2	630
Bull Terrier	M	M	2	3	4	25	4	7	4.25	9	330	273.0	350
Cane Corso	B	M	3	4	4	30	4	6.5	6	8	494	437	682.5
Caucasian Shepherd Dog	M	M	4	5	1	5	1	5	5	5	720	680	730
Cavalier King Charles Spaniel	B	M	2	2	10	46	8	4.5	3.25	5.75	230	210	252
Chihuahua	B	M	1	1	19	57	12	3	2	4	140	111.5	160
Chinese Crested Dog	M	M	2	1	10	31	3	3	2.25	3.75	155	118.8	176.2
Chow Chow	M	M	3	4	17	71	3	4	3	6	400	360	420
Cirneco dell’Etna	M	M	3	2	2	12	2	6	5.5	6.5	290	273.8	308.8
Epagneul Nain Continental Papillon	M	M	2	1	5	18	5	7	5	7	142	134.2	158
Czechoslovakian Wolfdog	M	M	3	4	3	20	3	7	6	7.5	390	362.2	410
Dalmatian	M	M	3	3	4	36	3	9	8	10	368	297.5	413
Deerhound	M	M	4	4	1	6	1	6	6	6	480	476.2	487.5
Dobermann	D	M	4	4	7	56	6	9	6.5	9	457	330	510
Dogue de Bordeaux	B	M	3	5	1	8	1	8	8	8	565	496.2	585
Drahthaar	M	M	3	4	1	9	1	9	9	9	308	294	329
Dachshund	M	A	1/2	2	14	59	11	4	3	5	173	146.5	217.5
English Cocker Spaniel	M	M	2	3	17	96	5	5	4	7	287.5	250	320
English Pointer	M	M	3	3	2	7	2	3.5	3.25	3.75	465	427	445
English Setter	M	M	3	3	5	32	2	6	4.75	7.5	389.5	340	427.2
Entlebucher Mountain Dog	M	M	3	3	1	7	1	7	7	7	344	336	357.5
Epagneul Breton	M	M	3	3	7	43	5	7	5	7	255	234	311.5
Fox Terrier Wire	M	M	2	2	2	6	2	3	2.5	3.5	225	215	237.2
German Shepherd dog	M	M	3	4	35	232	18	7	4.5	8.5	503	435	600
German Spitz Klein	M	M	2	1	1	2	2	3	2.5	3.5	125.5	125.2	125.8
German Spitz Zwerg-Pomeranian	M	M	2	1	3	11	1	4	3	4.5	124	114	150
Giant Schnauzer	M	M	3	4	1	10	1	10	10	10	357	315	370
Golden Retriever	M	M	3	4	19	148	10	8	5.5	10	235	228.8	245
Gordon Setter	M	M	3	4	2	16	2	8	7	9	406.5	388.8	428
Great Dane	M	M	4	5	5	44	5	10	9	11	647	512.5	698.2
Hovawart	M	M	3	4	8	68	3	9	7.75	9.25	560	500	590
Italian Greyhound	D	D	2	1	18	59	5	3	2	4	185	167.5	208
Italian Spinone	D	M	3	4	2	16	2	8	7.5	8.5	450	415	600
Jack Russel Terrier	M	M	2	2	15	67	8	5	3.5	5	200	180	220
Labrador Retriever	M	M	3	4	44	264	26	6	5	7.25	405.5	369.5	450
Lagotto	M	M	3	3	2	17	2	8.5	8.25	8.75	264	237	282
Lakeland Terrier	M	M	2	2	1	5	1	5	5	5	209	205	214
Leonberger	M	M	4	5	2	12	2	6	5	7	615	505	685
Little Lion Dog	M	M	2	2	1	4	1	4	4	4	190	190	190
Maltese	M	M	2	1	3	12	3	3	3	4.5	110.0	98.75	131.8
Maremma Sheepdog	M	M	4	4	3	23	2	7	6	9	595	491.2	688.5
Mastino Napoletano	B	M	4	5	3	21	2	8	5.5	9	790	609	912
Newfoundland	M	M	4	5	4	21	2	6	3.25	8	600	550	670
Poodle (miniature and toy)	M	M	2	1	6	12	5	2	1.25	2	116.5	99	155.5
Pug	B	B	2	2	8	32	4	3.5	2.75	5.5	164.5	135.5	192.8
Pumi	M	M	3	3	1	7	1	7	7	7	232	222	238
Rhodesian Ridgeback	M	M	4	4	7	76	3	12	8.5	12.5	390	358.5	420
Rottweiler	B	M	3	5	4	30	4	6.5	5.5	8.5	360	322.5	399.5
Saint Bernard dog	B	M	4	5	1	12	1	12	12	12	370	355	375
Samoiedo	M	M	3	3	1	7	1	7	7	7	229	218.8	248
Schapendoes	M	M	3	3	1	8	1	8	8	8	229	218.8	248
Rough Collie	D	M	3	3	1	1	1	1	1	1	155	155	155
Scottish Terrier	M	D	2	2	1	3	1	3	3	3	210	205	210
Segugio dell’Appennino	M	M	3	3	1	6	1	6	6	6	337	326	365.2
Shar Pei	M	M	3	3	3	11	3	3	2	5	450	425	475
Shetland Sheepdog	M	M	2	2	1	5	1	5	5	5	150	143	150
Shiba Inu	M	M	2	2	2	6	1	3	3	3	241	223.5	282.5
Shih Tzu	B	M	2	2	2	8	1	4	3.5	4.5	155	144.5	178
Siberian Husky	M	M	3	3	3	15	3	5	4.5	5.5	556	497.5	593.5
Staffordshire Bull Terrier	B	M	2	3	10	50	6	5	4	6	319.5	297.2	463.5
Standard Schnauzer	M	M	3	3	3	30	2	11	9.5	11	280	262.5	297.5
Tibetan Mastiff	M	M	3	5	15	107	2	7	6	8	450	385	490
Tibetan Terrier	M	M	2	2	1	2	1	2	2	2	217	215.5	218.5
Vizsla	M	M	3	3	1	6	1	6	6	6	400	4000	437.5
Volpino Italiano	M	M	2	1	6	22	4	4	3.25	4	132.5	85	173.8
Weimaraner	M	M	3	4	4	25	3	6.5	3.75	9	450	410	495
West Highland White T.	M	M	2	2	16	68	7	4	3	5.25	180	150	200
Whippet	D	D	3	3	2	13	2	6.5	6.25	6.75	352	332	380
White Swiss Shepherd dog	M	M	3	4	3	22	2	8	7	8	352	323.5	460
Yorkshire Terrier	M	M	1	1	5	14	3	3	2	4	120	106.2	128.8
Zwergpinscher	M	M	2	1	6	24	4	4	3	5.75	145	123	176.8
Zwergschnauzer	M	M	2	2	10	40	6	4	3.25	4	184.5	165.5	193.5

^a^ Head shape: B = brachycephalic; M = mesocephalic; D = dolichocephalic; ^b^ body shape: B = brachymorph; M = mesomorph; D = dolichomorph; A = anacholicomorph; ^c^ size: 1 = toy; 2 = small; 3 = medium; 4 = large; ^d^ BW means maternal body weight: 1 = <5 kg; 2 = 5 ≤ BW ≤ 10 kg; 3 = 10 < BW ≤ 25 kg; 4 = 25 < BW ≤ 45 kg; 5 = >45 kg; ^e^ N_L_ means number of litters; ^f^ N_P_ means number of pups; ^g^ N_K_ means number of kennels; ^h^ bBW means body weight of pups at birth (grams); ^I^ Q1 means first quartile; ^l^ Q3 means third quartile.

**Table 2:** Combinations of maternal morphotype, size and body weight and the corresponding pup number and birth weight in our canine sample.

Head Shape ^a^	Body Shape ^b^	Size ^c^	BW ^d^	N_L_^e^	N_P_^f^	N_p_						bBW ^g^					
						Min	Q1 ^h^	Median	Mean	Q3 ^i^	Max	Min	Q1 ^h^	Median	Mean	Q3 ^i^	Max
B	B	2	2	8	32	1	2.75	3.5	4	5.5	7	86	135.5	164.5	161.5	192.8	230
B	B	2	3	18	76	1	3	4	4.33	6	7	55	176	241.5	243.9	288.2	510
B	M	1	1	19	56	1	2	3	3	4	5	60	111.5	140	134.8	160	207
B	M	2	1	2	11	4	4.75	5.5	5.5	6.25	7	85	117	136	163	227.5	260
B	M	2	2	20	74	1	2.75	3	3.7	5	8	120	176.5	220	211.3	245	306
B	M	2	3	10	50	3	4	5	5	6	7	234	297.2	319.5	394.9	463.5	766
B	M	3	4	16	108	2	5	7.5	7	9	11	315	409	455.5	493.3	554	900
B	M	3	5	5	38	4	6	7	7.6	8	13	248	333.8	375	396.8	436.2	620
B	M	4	5	7	55	3	6	8	7.86	10	12	500	580	620	677.1	692	1250
M	M	1	1	5	14	1	2	3	2.8	4	4	90	106.2	120	129.6	128.8	202
M	M	2	1	51	182	1	2	3	3.51	5	7	44	114	142	147.8	179	266
M	M	2	2	50	208	2	3	4	4.16	5	7	90	167.8	190	189.6	214.2	350
M	M	2	3	28	166	2	5	6	5.93	7	9	130	247.8	286	287.5	330	500
M	M	3	2	2	12	5	5.5	6	6	6.5	7	235	273.8	290	289.2	308.8	335
M	M	3	3	60	392	1	5	7	6.55	8	11	148	280	344.5	341.1	392	650
M	M	3	4	190	1203	1	4	7	6.37	8	13	57	400	450	464.3	530	900
M	M	3	5	15	105	4	6	7	7.13	8	10	220	390	450	443	490	650
M	M	4	4	13	117	5	7	8	9.15	12	15	220	370	420	459.5	530	770
M	M	4	5	13	84	1	4	8	6.92	9	11	200	507.5	612.5	594	690	900
M	A	1	2	12	47	1	2.75	4	3.92	5	7	116	144.5	160	170.6	187.5	250
M	A	2	2	3	15	3	3.5	4	5	6	8	200	210	230	237.1	258	305
M	A	2	3	7	51	4	5.5	7	7.29	9	11	40	350	443	413.8	501	580
D	M	3	3	1	1	1	1	1	1	1	1	155	155	155	155	155	155
D	M	3	4	2	16	7	7.5	8	8	8.5	9	400	415	450	511.2	600	700
D	M	4	4	8	59	3	6	8	7.75	9	13	60	340	471	434.1	530.5	780
D	D	2	1	18	59	2	2	3	3.28	4	7	116	167.5	185	188.8	208	281
D	D	3	3	2	13	6	6.25	6.5	6.5	6.75	7	205	332	352	347.4	380	417
D	D	4	3	2	11	5	5.5	6	6	6.5	7	420	480	500	516.4	562.5	600
D	D	4	4	1	11	11	11	11	11	11	11	370	390.5	446	438.5	480.5	500

^a^ Head shape: B = brachycephalic; M = mesocephalic; D = dolichocephalic; ^b^ body shape: B = brachymorph; M = mesomorph; D = dolichomorph; A = anacholicomorph; ^c^ size: 1 = toy; 2 = small; 3 = medium; 4 = large; ^d^ BW means maternal body weight: 1 = <5 kg; 2 = 5 ≤ BW ≤ 10 kg; 3 = 10 < BW ≤ 25 kg; 4 = 25 < BW ≤ 45 kg; 5 = >45 kg; ^e^ N_L_ means number of litters; ^f^ N_P_ means number of pups; ^g^ bBW means body weight of pups at birth (grams); ^h^ Q1 means first quartile; ^i^ Q3 means third quartile.

**Table 3:** Birth weight of pups and maternal characteristics: results of linear mixed regression model univariate analysis.

Variable	Mean ^f^	Contrast	Mean Ratio	95% Lower Limit	95% Upper Limit	*t*	*p*-Value
Head shape ^a^	264.36	reference					
	323.37	M/B	1.22	1.10	1.37	3.57	<0.001
	269.81	D/B	1.02	0.83	1.25	0.20	0.8425
Body shape ^b^	221.32	reference					
	322.08	M/B	1.46	1.19	1.78	3.62	<0.001
	222.16	D/B	1.00	0.75	1.34	0.03	0.9794
	238.34	A/B	1.08	0.80	1.44	0.50	0.6197
Size ^c^	146.1	reference					
	203.22	2/1	1.39	1.24	1.56	5.52	<0.001
	423.99	3/1	2.90	2.59	3.26	18.17	<0.001
	519.26	4/1	3.55	3.07	4.11	17.11	<0.001
BW ^d^	147.39	reference					
	192.46	2/1	1.31	1.21	1.41	6.58	<0.001
	317.54	3/1	2.15	2.00	2.32	20.38	<0.001
	455.82	4/1	3.09	2.89	3.31	33.22	<0.001
	512.14	5/1	3.47	3.14	3.85	23.96	<0.001
Litter size *	221.91	reference one-pup increase	1.07	1.06	1.09	9.39	<0.001
Sex of pups ^e^	303.14	reference					
	314.91	1/0	1.04	1.03	1.05	7.30	<0.001
	260.66	2/0	0.86	0.80	0.92	−4.31	<0.001

* When the number of pups is adjusted for maternal size, the estimates for a one-pup increase per litter are: mean ratio = 0.99 (95% confidence limits: 0.98–1.01) t = −1.03 *p* value = 0.3024; ^a^ head shape: B = brachycephalic; M = mesocephalic; D = dolichocephalic; ^b^ Body shape: B = brachymorph; M = mesomorph; D = dolichomorph; A = anacholicomorph; ^c^ size: 1 = toy; 2 = small; 3 = medium; 4 = large; ^d^ BW means maternal body weight: 1 = <5 kg; 2 = 5 kg ≤ BW ≤ 10 kg; 3 = 10 kg < BW ≤ 25 kg; 4 = 25 kg < BW ≤ 45 kg; 5 = >45 kg; the category coded 1 is the reference; ^e^ sex of pups: 0 = female; 1 = male; 2 = unidentified; the category coded 0 is the reference; ^f^ mean (grams) is the estimated geometric mean of the weight distribution.

**Table 4:** Birth weight of pups and maternal characteristics: results of final linear mixed regression model multivariable analysis (step-wise selection procedure); body shape and weight, number of newborns, sex of pups.

Variable	Contrast	Mean Ratio ^d^	95% Lower Limit	95% Upper Limit	*t*	*p*-Value
Body shape ^a^	M/B	1.29	1.16	1.45	4.57	<0.001
	D/B	1.69	1.44	1.98	6.41	<0.001
	A/B	1.36	1.16	1.58	3.88	<0.001
BW ^b^	2/1	1.41	1.3	1.54	8.23	<0.001
	3/1	2.45	2.26	2.65	22.1	<0.001
	4/1	3.48	3.23	3.75	32.82	<0.001
	5/1	3.98	3.58	4.43	25.36	<0.001
Litter size	one-pup increase	0.98	0.97	0.99	−4.36	<0.001
Sex of pups ^c^	1/0	1.04	1.03	1.05	7.13	<0.001
	2/0	0.85	0.80	0.91	−4.6	<0.001

^a^ Body shape: B = brachymorph; M = mesomorph; D = dolichomorph; A = anacholicomorph; ^b^ BW means maternal body weight: 1 = <5 kg; 2 = 5 kg ≤ BW ≤ 10 kg; 3 = 10 kg < BW ≤ 25 kg; 4 = 25 kg < BW ≤ 45 kg; 5 = >45 kg; the category coded 1 is the reference; ^c^ sex of pups: 0 = female; 1 = male; 2 = unidentified; the category coded 0 is the reference; ^d^ mean ratio means model estimated ratio between geometric means.

**Table 5:** Birth weight of pups and maternal characteristics: results of final linear mixed regression model multivariable analysis (step-wise selection procedure); head and body shape, size, number of newborns, sex of pups.

Variable	Contrast	Mean Ratio ^e^	95% Lower Limit	95% Upper Limit	*t*	*p*-Value
Head shape ^a^	M/B	0.8	0.74	0.87	−5.42	<0.001
	D/B	0.66	0.53	0.81	−3.95	<0.001
Body shape ^b^	M/B	1.07	0.93	1.24	0.96	0.3377
	D/B	1.24	0.95	1.63	1.57	0.1163
	A/B	1.87	1.51	2.32	5.68	<0.001
Size ^c^	2/1	1.78	1.56	2.02	8.83	<0.001
	3/1	4.04	3.52	4.63	20.03	<0.001
	4/1	5.15	4.36	6.09	19.22	<0.001
Litter size	one-pup increase	0.99	0.98	1	−2.21	0.0275
Sex of pups ^d^	1/0	1.04	1.03	1.05	7.08	<0.001
	2/0	0.85	0.80	0.91	−4.57	<0.001

^a^ Head shape: B = brachycephalic; M = mesocephalic; D = dolichocephalic; ^b^ body shape: B = brachymorph; M = mesomorph; D = dolichomorph; A = anacholicomorph; ^c^ size: 1 = toy; 2 = small; 3 = medium; 4 = large. The category coded 1 is the reference; ^d^ sex of pups: 0 = female; 1 = male; 2 = unidentified; the category coded 0 is the reference; ^e^ mean ratio means model estimated ratio between geometric means.

**Table 6:** Number of pups and maternal characteristics: results of Poisson’s regression model univariate analysis.

Variable	Mean ^e^	Contrast	Mean Ratio	95% Lower Limit	95% Upper Limit	Wald Statistics	*p*-Value
Head shape ^a^	4.829	reference					
	5.817	M/B	1.205	1.095	1.325	3.839	<0.0001
	5.118	D/B	1.060	0.892	1.259	0.662	0.5080
Body shape ^b^	4.231	reference					
	5.754	M/B	1.360	1.124	1.645	3.168	0.0015
	4.130	D/B	0.976	0.742	1.285	−0.171	0.8642
	5.136	A/B	1.214	0.934	1.579	1.448	0.1476
Size ^c^	3.278	reference					
	4.293	2/1	1.310	1.081	1.586	2.76	0.00578
	6.495	3/1	1.981	1.645	2.386	7.208	<0.0001
	7.932	4/1	2.420	1.964	2.982	8.299	<0.0001
BW ^d^	3.368	reference					
	4.084	2/1	1.212	1.046	1.406	2.552	0.0107
	5.969	3/1	1.772	1.555	2.019	8.592	<0.0001
	6.657	4/1	1.976	1.752	2.229	11.082	<0.0001
	7.250	5/1	2.152	1.836	2.523	9.455	<0.0001

^a^ Head shape: B = brachycephalic; M = mesocephalic; D = dolichocephalic; ^b^ body shape: B = brachymorph; M = mesomorph; D = dolichomorph; A = anacholicomorph; ^c^ size: 1 = toy; 2 = small; 3 = medium; 4 = large; ^d^ BW means maternal body weight: 1 = <5 kg; 2 = 5 kg ≤ BW ≤ 10 kg; 3 = 10 kg < BW ≤ 25 kg; 4 = 25 kg < BW ≤ 45 kg; 5 = >45 kg; the category coded 1 is the reference; ^e^ mean is expressed in grams.

**Table 7:** Litter size and maternal characteristics: results of final Poisson regression model multivariable analysis (step-wise selection procedure); body shape and weight.

Variable	Contrast	Mean Ratio	95% Lower limit	95% Upper Limit	Wald Statistics	*p*-Value
Body shape ^a^	M/B	1.303	1.069	1.588	2.619	0.0088
	D/B	1.355	1.015	1.808	2.061	0.0393
	A/B	1.407	1.078	1.837	2.514	0.0119
BW ^b^	2/1	1.23	1.05	1.441	2.567	0.0103
	3/1	1.835	1.599	2.105	8.66	<0.001
	4/1	1.991	1.753	2.261	10.593	<0.001
	5/1	2.169	1.84	2.556	9.225	<0.001

^a^ Body shape: B = brachymorph; M = mesomorph; D = dolichomorph; A = anacholicomorph; ^b^ BW means maternal body weight: 1 = <5 kg; 2 = 5 kg ≤ BW ≤ 10 kg; 3 = 10 kg < BW ≤ 25 kg; 4 = 25 kg < BW ≤ 45 kg; 5 = >45 kg; the category coded 1 is the reference.

**Table 8:** Litter size and maternal characteristics: results of final Poisson regression model multivariable analysis (step-wise selection procedure); body shape and size.

Variable	Contrast	Mean Ratio	95% Lower Limit	95% Upper Limit	Wald Statistic	*p*-Value
Body shape ^a^	M/B	1.007	0.824	1.231	0.07	0.9442
	D/B	0.841	0.638	1.109	−1.228	0.2194
	A/B	1.487	1.126	1.963	2.795	0.0052
Size ^b^	2/1	1.506	1.225	1.852	3.883	<0.001
	3/1	2.298	1.87	2.825	7.906	<0.001
	4/1	2.836	2.26	3.559	8.995	<0.001

^a^ Body shape: B = brachymorph; M = mesomorph; D = dolichomorph; A = anacholicomorph; ^b^ size: 1 = toy; 2 = small; 3 = medium; 4 = large; the category coded 1 is the reference.

**Table 9:** Number and birth weight of pups per litter in five breeds.

Breed	N_L_^a^	N_P_^b^	N_P_^b^						bBW ^c^					
			Min	Q1 ^d^	Median	Mean	Q3 ^e^	Max	Min	Q1 ^d^	Median	Mean	Q3 ^e^	Max
German Shepherd	35	232	2	4.5	7	6.629	8.5	12	57	435	503	510.5	600	900
Golden Retriever	19	148	3	5.5	8	7.789	10	13	280	426.2	461.5	475.1	527.5	750
Labrador Retriever	44	264	1	5	6	6	7.25	10	188	369.5	405.5	412.1	450	624
Jack Russel Terrier	15	67	2	3.5	5	4.467	5	7	122	180	200	201.3	220	320
WHWT ^f^	16	68	2	3	4	4.25	5.25	6	90	150	180	175	200	280

^a^ N_L_ = number of litters; ^b^ N_P_ = number of pups; ^c^ bBW = birth weight of pups (grams); ^d^ Q1 = first quartile; ^e^ Q3 = third quartile; ^f^ WHWT = West Highland White Terrier.

## Supplementary Materials

Supplementary File 1

## Appendix A

**Table A1:** Number of Dogs Registered in the Genealogical Book ENCI from 1 January 2015–31 December 2015.

Breed	Number
Affenpinscher	13
Afghan Hound	81
Airedale Terrier	63
Akita Inu	1283
Alaskan Malamute	562
Alpenlaendische Dachsbracke	536
American Akita	461
American Cocker	87
American Staffordshire T.	4484
Anatolian Shepherd	26
Anglo Francais De Petite Venerie	66
Appenzeller Mountain dog	48
Argentine Dogo	1124
Ariegeois	663
Australian Cattle Dog	396
Australian Kelpie	39
Australian Shepherd	1567
Australian Silky Terrier	7
Azawakh	8
Basenji	60
Basset Fauve De Bretagne	16
Basset hound	338
Beagle	1402
Beagle Harrier	79
Bearded Collie	49
Beauceron	132
Bedlington Terrier	15
Belgian Shepherd Dog	886
Bergamasco Shepherd Dog	63
Bernese Mountain Dog	1554
Bichon A Poil Frise	232
Bichon Havanais	75
Black Russian Terrier	41
Bloodhound	86
Bobtail	28
Bolognese	358
Border Collie	3135
Border Terrier	13
Borzoi	89
Boston Terrier	338
Bouledogue	1822
Bouvier des Flandres	22
Boxer	3682
Bracco Italiano	694
Braque d'Auvergne	1
Braque français	172
Brazilian Mastiff	35
Briard	58
Briquet Griffon Vendeen	654
Broholmer	4
Brussel Griffon	31
Bull Terrier	516
Bulldog	2153
Bullmastiff	346
Byerischer gebirgsschweisshund	179
Cairn Terrier	39
Canaan Dog	14
Cane Corso	3957
Cao De Agua	32
Cao De Castro Laboreiro	3
Catalan Shepherd Dog	10
Caucasian Shepherd Dog	418
Cavalier King Charles Spaniel	1313
Central Asian Shepherd Dog	394
Chesapeake Bay Retriever	10
Chihuahua	5794
Chin	58
Chinese Crested Dog	74
Chow Chow	179
Cirneco dell’Etna	105
Clumber Spaniel	59
Coton De Tulear	99
Czechoslovakian Wolfdog	1362
Dachshund	2904
Dalmatian	146
Deerhound	9
Dobermann	1693
Dogo Canario	73
Dogue De Bordeaux	801
Dutch Shepherd Dog	33
English Cocker Spaniel	2084
English Pointer	2339
English Setter	13,702
English Springer Spaniel	1773
Entlebucher Mountain Dog	13
Epagneul Breton	3275
Epagneul Nain Continental Papillon	108
Erdélyi Kopó	14
Eurasier	25
Flat Coated Retriever	205
Fox Terrier Wire	181
Galgo Espanol	1
Gascon Saintongeois	194
German Jagdterrier	176
German Shepherd	14,369
German Shorthaired Pointer	2435
German Spaniel	59
German Spitz	905
German Wirehaired Pointer	763
Giant Schnauzer	339
Golden Retriever	5692
Gordon Setter	357
Grand Griffon Vendeen	2
Great Dane	1075
Greyhound	50
Griffon Belge	13
Griffon Bleu De Gascogne	319
Griffon Nivernais	42
Hannoverischer Schweisshund	83
Hokkaido	5
Hound of the Maremma	2923
Hovawart	232
Hungarian Vizsla SH	259
Irish Soft- Coated Wheaten Terrier	43
Irish Terrier	31
Irish Water Spaniel	1
Irish Wolfhound	24
Istrian Hound Rough Hair	19
Istrian Hound Short Hair	212
Italian Greyhound	295
Italian Hound Rough Haired	1070
Italian Hound Smooth Haired	3570
Italian Spinone	506
Jack Russel Terrier	5257
Japanese Spitz	26
Karelian Bear Dog	39
Kerry Blue Terrier	25
King Charles Spaniel	8
Komondor	3
Kooikerhondje	5
Labrador Retriever	9414
Lagotto Romagnolo	2341
Lakeland Terrier	98
Landseer	14
Lappinkoira	16
Leonberger	161
Lhasa Apso	129
Little Lion Dog	2
Maltese	1631
Manchester Terrier	16
Maremma and the Abruzzes Sheepdog	993
Mastiff	32
Miniature English Bull Terrier	222
Mudi	7
Neapolitan Mastiff	514
Newfoundland	406
Norfolk Terrier	30
Norwich Terrier	20
Nova Scotia Duck Tolling Retriever	61
Parson Russell Terrier	174
Pekingese	17
Perdigueiro Português	1
Petit Basset Griffon Vendeen	56
Petit Bleu De Gascogne	148
Petit Brabançon	9
Pharaon Hound	1
Picardy Shepherd	2
Pinscher	23
Podenco Ibicenco	6
Podengo Portugues	1
Polish Greyhound	1
Polish Lowland Sheepdog	5
Poodle	2072
Porcelaine	153
Posavatz Hound	48
Pug	632
Puli	1
Pumi	2
Pyrenean Mastiff	91
Pyrenean Mountain Dog	115
Pyrenean Shepherd	14
Rhodesian Ridgeback	318
Romanian Shepherd Bucovina	10
Romanian Shepherd Dog Mioritic	27
Rottweiler	4080
Rough Collie	391
Russian Toy	16
Saarloos Wolfdog	48
Saint Bernard Dog	629
Saluki	34
Samoiedo	304
Schapendoes	16
Schipperke	13
Scottish Terrier	113
Sealyham Terrier	5
Segugio dell’Appennino	197
Segugio Maremmano	2923
Serbian Hound	1
Serbian Tricolour hound	7
Shar Pei	551
Shetland Sheepdog	168
Shiba Inu	701
Shih Tzu	604
Shikoku	9
Siberian Husky	857
Skye Terrier	12
Sloughi	1
Slovakian hound	103
Smooth Collie	1
Smooth Fox Terrier	80
Spanish Mastiff	11
Staffordshire Bull Terrier	1266
Standard Schnauzer	208
Swiss hound	248
Swiss Mountain Dog	86
Tibetan Mastiff	124
Tibetan Spaniel	5
Tibetan Terrier	48
Tosa	2
Volpino Italiano	130
Weimaraner	1158
Welsh Corgi Cardigan	2
Welsh Corgi Pembroke	195
Welsh Springer Spaniel	3
Welsh Terrier	36
West Highland White T.	592
Whippet	489
White Swiss Shepherd Dog	438
Xoloitzcuintle	1
Yorkshire Terrier	551
Yugoslavian Shepherd Dog	25
Zwergpinscher	454
Zwergschnauzer	785
**Total**	154,195
