# Supplementary material for: A National Census of Birth Weight in Purebred Dogs in Italy
Source: Animals (Basel). 2017 May 30;7(6):43. doi: 10.3390/ani7060043 (PMC5483606; doi:10.3390/ani7060043)
Supplement: Supplementary File 1 [file animals-07-00043-s001.docx]

**Appendix 1.** Number of dogs registered in the genealogical book ENCI from 1^st^ January 2015 to 31^st^ December 2015.

| **Breed** | **Number** |
| --- | --- |
| Affenpinscher | 13 |
| Afgan Hound | 81 |
| Airedale Terrier | 63 |
| Akita Inu | 1283 |
| Alaskan Malamute | 562 |
| Alpenlaendische Dachsbracke | 536 |
| American Akita | 461 |
| American Cocker | 87 |
| American Staffordshire T. | 4484 |
| Anatolian Shepherd | 26 |
| Anglo Francais De Petite Venerie | 66 |
| Appenzeller Mountain dog | 48 |
| Argentine Dogo | 1124 |
| Ariegeois | 663 |
| Australian Cattledog | 396 |
| Australian Kelpie | 39 |
| Australian Shepherd | 1567 |
| Australian Silky Terrier | 7 |
| Azawakh | 8 |
| Basenji | 60 |
| Basset Fauve De Bretagne | 16 |
| Bassethound | 338 |
| Beagle | 1402 |
| Beagle Harrier | 79 |
| Bearded Collie | 49 |
| Beauceron | 132 |
| Bedlington Terrier | 15 |
| Belgian Shepherd dog | 886 |
| Bergamasco Shepherd Dog | 63 |
| Bernese Mountain dog | 1554 |
| Bichon A Poil Frise | 232 |
| Bichon Havanais | 75 |
| Black Russian Terrier | 41 |
| Bloodhound | 86 |
| Bobtail | 28 |
| Bolognese | 358 |
| Border Collie | 3135 |
| Border Terrier | 13 |
| Borzoi | 89 |
| Boston Terrier | 338 |
| Bouledogue | 1822 |
| Bouvier des Flandres | 22 |
| Boxer | 3682 |
| Bracco Italiano | 694 |
| Braque d'Auvergne | 1 |
| Braque français | 172 |
| Brazilian Mastiff | 35 |
| Briard | 58 |
| Briquet Griffon Vendeen | 654 |
| Broholmer | 4 |
| Brussel Griffon | 31 |
| Bull Terrier | 516 |
| Bulldog | 2153 |
| Bullmastiff | 346 |
| Byerischer gebirgsschweisshund | 179 |
| Cairn Terrier | 39 |
| Canaan Dog | 14 |
| Cane Corso | 3957 |
| Cao De Agua | 32 |
| Cao De Castro Laboreiro | 3 |
| Catalan Sheepherd Dog | 10 |
| Caucasian Shepherd Dog | 418 |
| Cavalier King Charles Spaniel | 1313 |
| Central Asian Shepherd Dog | 394 |
| Chesapeake Bay Retriever | 10 |
| Chihuahua | 5794 |
| Chin | 58 |
| Chinese crested dog | 74 |
| Chow Chow | 179 |
| Cirneco dell’Etna | 105 |
| Clumber Spaniel | 59 |
| Coton De Tulear | 99 |
| Czechoslovakian Wolfdog | 1362 |
| Dachshund | 2904 |
| Dalmatian | 146 |
| Deerhound | 9 |
| Dobermann | 1693 |
| Dogo Canario | 73 |
| Dogue De Bordeaux | 801 |
| Dutch Shepherd Dog | 33 |
| English Cocker Spaniel | 2084 |
| English Pointer | 2339 |
| English Setter | 13702 |
| English Springer Spaniel | 1773 |
| Entlebucher Mountain Dog | 13 |
| Epagneul Breton | 3275 |
| Epagneul Nain Continental Papillon | 108 |
| Erdélyi Kopó | 14 |
| Eurasier | 25 |
| Flat Coated Retriever | 205 |
| Fox Terrier Wire | 181 |
| Galgo Espanol | 1 |
| Gascon Saintongeois | 194 |
| German Jagdterrier | 176 |
| German Shepherd | 14369 |
| German Shorthaired Pointer | 2435 |
| German Spaniel | 59 |
| German Spitz | 905 |
| German Wirehaired Pointer | 763 |
| Giant Schnauzer | 339 |
| Golden Retriever | 5692 |
| Gordon Setter | 357 |
| Grand Griffon Vendeen | 2 |
| Great Dane | 1075 |
| Greyhound | 50 |
| Griffon Belge | 13 |
| Griffon Bleu De Gascogne | 319 |
| Griffon Nivernais | 42 |
| Hannoverischer Schweisshund | 83 |
| Hokkaido | 5 |
| Hound of the Maremma | 2923 |
| Hovawart | 232 |
| Hungarian Vizsla SH | 259 |
| Irish Soft- Coated Wheaten Terrier | 43 |
| Irish Terrier | 31 |
| Irish Water Spaniel | 1 |
| Irish Wolfhound | 24 |
| Istrian Hound Rough Hair | 19 |
| Istrian Hound Short Hair | 212 |
| Italian Greyhound | 295 |
| Italian Hound Rough Haired | 1070 |
| Italian Hound Smooth Haired | 3570 |
| Italian Spinone | 506 |
| Jack Russel Terrier | 5257 |
| Japanese Spitz | 26 |
| Karelian Bear Dog | 39 |
| Kerry Blue Terrier | 25 |
| King Charles Spaniel | 8 |
| Komondor | 3 |
| Kooikerhondje | 5 |
| Labrador Retriever | 9414 |
| Lagotto Romagnolo | 2341 |
| Lakeland Terrier | 98 |
| Landseer | 14 |
| Lappinkoira | 16 |
| Leonberger | 161 |
| Lhasa Apso | 129 |
| Little Lion Dog | 2 |
| Maltese | 1631 |
| Manchester Terrier | 16 |
| Maremma and the Abruzzes Sheepdog | 993 |
| Mastiff | 32 |
| Miniature English Bull Terrier | 222 |
| Mudi | 7 |
| Neapolitan Mastiff | 514 |
| Newfoundland | 406 |
| Norfolk Terrier | 30 |
| Norwich Terrier | 20 |
| Nova Scotia Duck Tolling Retriever | 61 |
| Parson Russell Terrier | 174 |
| Pekingese | 17 |
| Perdigueiro Português | 1 |
| Petit Basset Griffon Vendeen | 56 |
| Petit Bleu De Gascogne | 148 |
| Petit Brabançon | 9 |
| Pharaon Hound | 1 |
| Picardy Shepherd | 2 |
| Pinscher | 23 |
| Podenco Ibicenco | 6 |
| Podengo Portugues | 1 |
| Polish Greyhound | 1 |
| Polish Lowland Sheepdog | 5 |
| Poodle | 2072 |
| Porcelaine | 153 |
| Posavatz Hound | 48 |
| Pug | 632 |
| Puli | 1 |
| Pumi | 2 |
| Pyrenean Mastiff | 91 |
| Pyrenean Mountain Dog | 115 |
| Pyrenean Shepherd | 14 |
| Rhodesian Ridgeback | 318 |
| Romanian Shepherd Bucovina | 10 |
| Romanian Shepherd Dog Mioritic | 27 |
| Rottweiler | 4080 |
| Rough Collie | 391 |
| Russian Toy | 16 |
| Saarloos Wolfdog | 48 |
| Saint Bernard Dog | 629 |
| Saluki | 34 |
| Samoiedo | 304 |
| Schapendoes | 16 |
| Schipperke | 13 |
| Scottish Terrier | 113 |
| Sealyham Terrier | 5 |
| Segugio dell’Appennino | 197 |
| Segugio Maremmano | 2923 |
| Serbian Hound | 1 |
| Serbian Tricolour hound | 7 |
| Shar Pei | 551 |
| Shetland Sheepdog | 168 |
| Shiba Inu | 701 |
| Shih Tzu | 604 |
| Shikoku | 9 |
| Siberian Husky | 857 |
| Skye Terrier | 12 |
| Sloughi | 1 |
| Slovakian hound | 103 |
| Smooth Collie | 1 |
| Smooth Fox Terrier | 80 |
| Spanish Mastiff | 11 |
| Staffordshire Bull Terrier | 1266 |
| Standard Schnauzer | 208 |
| Swiss hound | 248 |
| Swiss Mountain Dog | 86 |
| Tibetan Mastiff | 124 |
| Tibetan Spaniel | 5 |
| Tibetan Terrier | 48 |
| Tosa | 2 |
| Volpino Italiano | 130 |
| Weimaraner | 1158 |
| Welsh Corgi Cardigan | 2 |
| Welsh Corgi Pembroke | 195 |
| Welsh Springer Spaniel | 3 |
| Welsh Terrier | 36 |
| West Highland White T. | 592 |
| Whippet | 489 |
| White Swiss Shepherd dog | 438 |
| Xoloitzcuintle | 1 |
| Yorkshire Terrier | 551 |
| Yugoslavian Shepherd Dog | 25 |
| Zwergpinscher | 454 |
| Zwergschnauzer | 785 |
|  |  |
| **Total** | 154195 |
